# In Nature's School

**Published:** 1889-09-07

**Authors:** 


### The Doctor's Pulpit.

### IN NATURE'S SCHOOL.

### Closed Records.

Nature's day ends with darkness, and for the most part with cessation of toil. When the night falls the day's record is closed and can never be reopened. To the plants and the flowers, and probably also, as a rule, to the birds and the beasts, when the day is over its labours, its sorrows, and its joys are as completely over and done with as if they had never been. To man almost exclusively is it given to remember sorrow or joy. Memory of the past is often a painful, though sometimes a pleasurable, endowment, and requires, like all other human endowments, to be kept well under the control of reason. Life is not meant to be a sorrow, nor a regret, nor a failure; but a duty and a success. It is probable that it never can be a great success except where it is controlled by well-balanced and calm reason. It may seem a success, especially if parts of it be taken separately, without being well under the control of reason; and it may seem a failure in parts even when reason is enthroned in her most majestic calmness and strength. But life must be taken as a whole, and any particular human life which has not placed reason on its throne and kept her there as mistress from year to year must be pronounced in any large and comparative survey "a total failure."

Reason is a docile faculty, if it may properly be called a faculty; docile and industrious. It loves to learn. It loves object lessons better than dialectic. Argument may lead to truth: fact is truth. When a man tells another that he will be wise to close many of the records of the past; to close the book of memory on many subjects and not to open it again, except for very good reasons, the other may doubt the wisdom of the advice or the possibility of adopting it. But when a man shows his neighbour the birds perched on the lofty branches of the trees, resting quietly through the hours of darkness, with no apparent memory of either the joys or the sorrows of the past day, the neighbour cannot deny that they sleep soundly and wake fit for duty and happiness. He cannot fail also to see that it would sometimes be a great advantage if he too, like the birds, could close up the records of the day when the day was over, or so soon as his head was laid for the night upon the pillow.

It is undoubtedly at times a very great advantage to forget; to forget completely and permanently. Sometimes it is even a necessity if the reason is to be saved and the work of life to be done. Goëthe has often been charged with selfishness, because he hated to remember or to hold in his mind the ghosts of past griefs. But if a man attaches more value to his work than he does to himself, as every man should who has worthy work, he will gradually come to the conclusion that Goëthe was on the whole right. A man may debilitate not only his reason but his moral energy by a too protracted and too profound memory of past griefs or wrong-doings. "Close the books" is good advice; shut them, lock up the brass clasps, and put the volumes away where they shall never see the light again until they are needed for guidance in practical duty.

Nature closes her books not only occasionally, but daily, monthly, yearly. Every sunset is a closed book, every waning of the moon, every transit of spring into summer, of summer into golden autumn, and of autumn into winter's snows. The brain easily learns tricks, much more easily than the hand learns skill and cunning. The brain, as every physiologist knows, is the master learner; the hand is only a pupil and a servant of the brain. But let it never be forgotten that the brain was originally meant to be, and is, in every perfectly healthy person, the servant of what mental philosophers call the ego, the true and proper person. The brain, itself, we repeat, is the servant of the true person or soul. The brain learns tricks. When the tricks are so well learnt as to be practised independently of the will; in other words, when the person loses control of himself, that is the beginning of insanity. The ancients knew that very well. The most familiar trick of the uncontrolled brain in remote ages was ungovernable passion, anger, rage. So says the Latin primer, "*Ira furor brevis est.*" Most true, "Rage is brief madness." Nobody will question the desirability of being able to control the temper, not even the angry man himself. He who can shut the door on a rage or a fit of passion saves himself not only from much mental mortification, but from a very profitless expenditure of nervous energy, and so from a great waste of health.

Similarly, the brain learns tricks of memory, brings back again and again old sorrows, old failures, old losses, old mortifications; brings them back when they can serve no good purpose; brings them back when they are only injurious and miserable; brings them when the health is poor, when an exhausting day demands a sound night's sleep; brings them to "murder sleep" and to make night not only "hideous," but desperate. What would not the sleepless man give in the dead of night if he could banish at once from his memory the overwhelming bitterness of past grief? The brain has learnt the trick of it, and there are times when nothing whatever, not the most powerful drug in all the pharmacopœias of the world will lay the ghost of sorrow.

But that is disease. The brain that plays such tricks is not well in hand. It cannot spend its energy in that way without having all too little for its proper daily work. The door ought to be resolutely shut on such paralysing memories. If a man says he cannot shut the door, that it will fly open again and let out the ghosts in spite of him, then, we repeat, he is diseased. He needs treatment. He needs adequate treatment. It may be, probably is, rest of brain he requires. It may be, and probably also is, a forcible change of mental habit, only to be secured by a forcible change of physical surroundings. Perhaps he needs to escape from the peculiar ideas that hold him in thrall at the moment, and to compel his brain of necessity to spend its energy in other directions. Not rest alone, but change; and not change alone, but such a change as shall of necessity start off the brain on entirely new lines of activity, and keep it there.

Ought a man to think most of his life's work, or most of himself? That is a question which lies at the root of many other practical problems. The intelligent Christian moralist will say he should think most of his work; and the intelligent physician who has looked beneath the surface of things will say exactly the same when considering the subject solely from a health point of view. Work, entered into with all the heart and soul, and done as well as it is in the power of the worker to do it, is such a brain and nerve tonic as all the chemists' laboratories in the world cannot produce the equal of. Nothing locks the door of memory and bars it so fast and tight as honest work well done. Let a man give his soul to his work, let him find his pleasure in it, let him do it for its own sake, and stand to it until it is completed and finished, and he will not, as a rule, be troubled with ghosts at midnight who "murder sleep" and "urge to madness the despairing soul." "Let the dead bury their dead," said the greatest of all teachers. Let the paralysing memories of the past be driven away and forgotten. Present time and strength are for present duty, and present duty done brings present reward of mental wholesomeness and physical health.

